# Mitochondrial Dysfunction in Astrocytes Impairs the Generation of Reactive Astrocytes and Enhances Neuronal Cell Death in the Cortex Upon Photothrombotic Lesion

**DOI:** 10.3389/fnmol.2019.00040

**Published:** 2019-02-22

**Authors:** Christian Fiebig, Silke Keiner, Birgit Ebert, Iris Schäffner, Ravi Jagasia, D. Chichung Lie, Ruth Beckervordersandforth

**Affiliations:** ^1^Institute of Biochemistry, Emil Fischer Center, Friedrich-Alexander-Universität Erlangen-Nürnberg, Erlangen, Germany; ^2^Hans Berger Department of Neurology, Jena University Hospital, Jena, Germany; ^3^Institute of Developmental Genetics, Helmholtz Center Munich, German Research Center for Environmental Health, Munich, Germany; ^4^F. Hoffmann-La Roche, Ltd., CNS Discovery, Pharma Research and Early Development, Basel, Switzerland

**Keywords:** mitochondrial metabolism, astrocytes, stroke/photothrombotic lesion, electron transport chain, oxidative phosphorylation, reactive gliosis, Tfam

## Abstract

Mitochondria are key organelles in regulating the metabolic state of a cell. In the brain, mitochondrial oxidative metabolism is the prevailing mechanism for neurons to generate ATP. While it is firmly established that neuronal function is highly dependent on mitochondrial metabolism, it is less well-understood how astrocytes function rely on mitochondria. In this study, we investigate if astrocytes require a functional mitochondrial electron transport chain (ETC) and oxidative phosphorylation (oxPhos) under physiological and injury conditions. By immunohistochemistry we show that astrocytes expressed components of the ETC and oxPhos complexes *in vivo*. Genetic inhibition of mitochondrial transcription by conditional deletion of *mitochondrial transcription factor A* (*Tfam*) led to dysfunctional ETC and oxPhos activity, as indicated by aberrant mitochondrial swelling in astrocytes. Mitochondrial dysfunction did not impair survival of astrocytes, but caused a reactive gliosis in the cortex under physiological conditions. Photochemically initiated thrombosis induced ischemic stroke led to formation of hyperfused mitochondrial networks in reactive astrocytes of the perilesional area. Importantly, mitochondrial dysfunction significantly reduced the generation of new astrocytes and increased neuronal cell death in the perilesional area. These results indicate that astrocytes require a functional ETC and oxPhos machinery for proliferation and neuroprotection under injury conditions.

## Introduction

Astrocytes are highly abundant in the brain (Nedergaard et al., 2003) and central to homeostasis of the nervous system by, e.g., regulating glutamate, ion and water homeostasis, synapse formation and modulation, tissue repair, energy storage, and defense against oxidative stress (Volterra and Meldolesi, 2005; Belanger and Magistretti, 2009). Astrocytes are also critically important for brain metabolism (Hertz et al., 2007; Belanger et al., 2011). Bridging between neurons and blood vessels, astrocytes are major components of neurovascular coupling. Fine astrocytic processes cover synaptic contacts on one side (Iadecola and Nedergaard, 2007; Oberheim et al., 2009), while on the other side, astrocyte end-feet enwrap the brain microvasculature and regulate the vascular tone as well as blood–brain function (Attwell et al., 2010; Zhao et al., 2015). These morphological characteristics and a special regionalized molecular set-up allow astrocytes to sense neuronal activity at the synapse (via receptors for neurotransmitters, cytokines, growths factors, transporters, and ion channels), and react with the appropriate metabolic supply via their end-feet on the blood vessels (via glucose transporters and aquaporin 4), thereby coordinating synaptic needs and metabolic supply.

In contrast to neurons that sustain a high rate of oxidative mitochondrial metabolism, astrocytes characteristically perform glycolysis (Belanger et al., 2011). Still, astrocytes possess almost as many mitochondria as neurons (Lovatt et al., 2007), and transcriptome analysis indicated that astrocytes are equipped with the necessary molecular machinery to perform oxidative metabolism (Lovatt et al., 2007; Cahoy et al., 2008). However, it is an ongoing debate if and to which extent astrocytes *in vivo* perform and require oxPhos. A recent publication showed that conditional ablation of electron transport chain (ETC) neither affected long-term viability of astrocytes nor caused any obvious brain pathology (Supplie et al., 2017). An interesting question now is how astrocytes behave under stress conditions. Astrocytes have a unique capacity to adapt to conditions of metabolic challenge and are able to adjust their metabolic state to distinct injuries as assessed by transcriptome analysis (Hamby et al., 2012; Zamanian et al., 2012). Furthermore, preservation of mitochondrial respiratory function in astrocytes may be important for the brain’s energy balance and for production of antioxidants that contribute to neuronal protection (Greenamyre et al., 2003; Dugan and Kim-Han, 2004). In many neurodegenerative disorders and under injury conditions, the astrocyte’s response to injury and disease becomes increasingly recognized because astrocytes bare the potential to enhance neuronal survival and regeneration (Sofroniew and Vinters, 2010; Barreto et al., 2012).

Here, we investigated the impact of mitochondrial ETC and oxPhos in astrocytes *in vivo* under pathological conditions in a stroke model of photochemically initiated thrombosis (PIT). Toward this aim, we abolished ETC complexes I, III, and IV function as well as oxPhos complex V activity in astrocytes by conditional deletion of the mitochondrial transcription factor A (*Tfam*; Larsson et al., 1998). Deletion of *Tfam* did not impair survival of astrocytes but induced reactive gliosis in the cortex and led to morphological alteration of mitochondria in reactive astrocytes. Upon photothrombotic lesions, *Tfam*-deficiency worsened mitochondrial morphology phenotypes and impaired the generation of new astrocytes in the perilesional area. Most notably, dysfunctional mitochondrial respiration in astrocytes increased neuronal cell death in the perilesional area, indicating that astrocytes require functional mitochondrial machinery for proliferation after injury and for protecting the neurons from the damage induced by stroke.

## Materials and Methods

### Experimental Model and Subject Details

All experiments were carried out in accordance with the European Communities Council Directive (86/609/EEC). Animal experiments were approved by the Government of Upper Bavaria. For all experiments, mice were group housed in standard cages under a 12 h light/dark cycle with *ad libitum* access to water and food. The astrocyte specific conditional Tfam knockout line and the control line (*Tfam^cko^* and *Tfam^ctrl^*, respectively) were generated from *Tfam*^loxP/loxP^ mice (Larsson et al., 1998), GLAST::CreER^T2^ (Mori et al., 2006), CAG-CAT-EGFP reporter mice (Nakamura et al., 2006) and were described previously (Beckervordersandforth et al., 2017).

### Tamoxifen Administration

Tamoxifen was dissolved at 10 mg/ml in corn oil (Sigma) and animals were intraperitonially (i.p.) injected with 1 mg on postnatal days 14, 16, and 18 (Beckervordersandforth et al., 2017).

### Genotyping PCR

The following primers were used for genotyping: Tfam-A CTGCCTTCCTCTAGCCCGGG, Tfam-B GTAACAGCAGACAACTTGTG, Tfam-C CTCTGAAGCACATGGTCAAT. The expected size of PCR products for *Tfam^wt^* was 404 bp, for *Tfam*^*floxed*^ = 437 bp, and for *Tfam^cko^* = 329 bp.

### Astrocyte Culture

Primary astrocytes were isolated as previously described (Heinrich et al., 2010). Briefly, postnatal day 5 (P5) *CAG CAT GFP; Tfam^*fl/fl*^* mice were decapitated and cortices were dissected in ice-cold dissection medium (HBSS with Hepes 10 mM) by carefully removing all meninges. Dissected slices were minced into small tissue pieces, and further dissociated with a fire-polished Pasteur pipette into a single cell suspension. After centrifugation (900 rcf, 5 min, RT), the supernatant was discarded, and the pellet resuspended in 10 ml astrocyte medium (DMEM/F12, 0,45% Glucose, 10%FBS, 5% horse serum, B27, 10 ng/ml EFG and FGF). Cell suspension was transferred into in a medium sized flask (10 ml, 1T75) if two brains were pooled. Cells of one brain were transferred into a small flask (5 ml, 1T25). Cells were incubated at 37°C with 5% CO_2_. Medium was changed every 4 days after shaking (200 rpm) at room tempertaure (RT) to remove unattached tissue like microglia and oligodendrocytes. Cells were passaged by trypsination when cell density reached 70% confluence. For immunostainings, cells were seeded onto PDL-coated glass cover slips. Two days after passaging, cells were transduced with HTNCre protein (1, 2, or 4 μl). For immunochemistry, cells were fixed with 4% PFA for 5 min 6 days post-transduction. For PCR, cells were trypsinized 6 days post-transduction, and DNA was isolated using the QIAamp DNA Mini Kit (Qiagen).

### FACSorting

*Tfam^ctrl^* and *Tfam^cko^* animals were decapitated. The different brain areas were isolated and cut into small pieces. Tissue from one mouse was resuspended in 1 ml of enzyme mixture, incubated for maximal 25 min at 37°C, and dissociated with a fire-polished Pasteur pipette every 5 min. The enzyme mix contained 50 μl EDTA (50 mM), 50 μl L-Cystein (100 mM), 2 mg Papain, 5 mg Dispase, 2 mg DNAse I, and 167 μl MgSO_4_ dissolved in 5 ml HBSS, and was sterile filtrated before use. After digestion, the tissue-enzyme mix was passed through a 70 μm filter, and the filter was flushed with 2 ml DMEM/F12 (Gibco-32331) supplemented with 10% FBS. After centrifugation (1000 rcf for 3 min at RT), the pellet was resolved in 10 ml DMEM/F12 with 10% FCS. The centrifugation step was repeated and cell pellet was resuspended in 5 ml DMEM/F12 with 10% FCS mixed with 5 ml Percoll (4.5 ml Percoll in 0.5 ml 10x PBS) prior to 30 min centrifugation at 1000 rcf at RT. Then the cell pellet was washed with 1x PBS. After centrifugation (1000 rcf for 3 min at RT), the pellet was resuspended in 300 μl DMEM/F12 and passed through a 40 μm cell strainer. Recombined GFP^+^ astrocytes were FACSorted (BD FACS Aria in BD FACS Flow TM medium, with a sheath pressure of 70 psi and a nozzle diameter of 70 μm). DNA isolation was performed using the QIAamp DNA Mini Kit (Qiagen).

Genotyping PCR was performed to analyze recombination of the *Tfam* locus.

### Tissue Processing

Animals were sacrificed using CO_2_. Mice were transcardially perfused with 50 ml phosphate-buffered saline (PBS, pH 7.4) followed by 100 ml 4% paraformaldehyde (PFA) at a rate of 10 ml/min. Brains were post-fixed in 4% PFA for 12 h at 4°C and were subsequently transferred to a 30% sucrose solution. Coronal brain sections were produced using a sliding microtome (Leica Microsystems, Wetzlar, Germany) for phenotyping and morphological analysis.

### Histology and Counting Procedures

The following primary antibodies were used: chicken anti-GFP (1:2000, Aves), goat anti-HSP60 (1:500, Santa Cruz), mouse anti-OXPHOS/ETC mix (1:500, Abcam), mouse anti-NDUFB8 (1:500, Abcam), mouse anti-Cox1 (MTCO1; 1:500, Abcam), mouse anti-ATP5A (1:500, Abcam), mouse anti-GFAP (1:500, Sigma), rabbit anti-GFAP (1:500, DAKO), mouse anti-Nestin (1:500, Millipore), rat anti-BrdU (1:500, Bio-Rad, formerly Serotec); mouse anti-BrdU (1:500, Millipore), rabbit anti-Casp3 (1:100, Cell Signaling), mouse anti-NeuN (1:100, Merck Millipore), rabbit anti-Tfam (1:500, gift from Nils-Göran Larsson), mouse anti-CytC (1:500, Becton Dickson).

Primary antibodies were visualized with Alexa-conjugated secondary antibodies (all 1:400, Invitrogen). As negative controls staining were performed using secondary antibody only.

Immunofluorescent stainings were performed on free-floating 40 and 50 μm sections. Slices were washed three times with PBS and incubated with primary antibodies in PBS containing 0.5% Triton X-100 and 10% normal donkey serum (NDS) for 72 h at 4°C. After incubation with the primary antibody, tissue was thoroughly washed with PBS at room temperature and subsequently incubated with the secondary antibody in PBS containing 0.5% Triton X-100 and 10% NDS overnight at 4°C or 2 h at room temperature. After washing thoroughly in PBS, nuclei were stained with DAPI and sections were mounted on coverslips with Aqua poly mount (Polysciences).

For BrdU staining, tissue was pre-treated in 2 M HCL for 30 min, washed shortly in PBS, incubated in Borate buffer (0.1 M, pH 8.5) two times for 15 min, followed by washing with PBS before adding the BrdU antibody.

For immunostainings against activated Caspase 3 (Casp3) and mitochondrial markers HSP60, NDUFB8, Cox1, ATP5A, and mitochondrial complexes I–V mix (OXPHOS/ETC mix), sections were subjected to antigen retrieval. Slices were incubated in Tris-EDTA/Tween20 for 5 min at 99°C and washed three times with MilliQ water followed by one washing step with PBS prior incubation with primary antibody. Biotinylated secondary antibodies (1:400; Vector Laboratories) were used in combination with Alexa-conjugated to Streptavidin (1:400; Invitrogen) to enhance the signal of Casp3, NDUFB8, Cox1, HSP60, ATP5A, and OXPHOS/ETC mix.

Confocal single plane images and Z-stacks were taken at the Zeiss LSM 780 with four lasers (405, 488, 559, and 633 nm) and 40 and 63x objective lens. Images were processed using Fiji ImageJ and Adobe Photoshop CS5.

### Photochemically Initiated Thrombosis (PIT)

Photochemically initiated thrombosis was induced in 4 months-old *Tfam^ctrl^* (*n* = 6) and *Tfam^cko^* mice (*n* = 7), the time point when the mitochondrial phenotype of GlastCre::ER^T2^-mediated *Tfam* deletion manifested (Beckervordersandforth et al., 2017). For that mice were deeply anesthetized by intraperitonial (i.p.) injection of Fentanyl (0.05 mg/kg; Janssen-Cilag AG, New Brunswick, NJ, United States), Midazolam (5 mg/kg; Dormicum, Hoffmann-La Roche, Basel, Switzerland) and Medetomidine (0.5 mg/kg; Domitor, Pfizer, Inc., New York City, NY, United States) dissolved in 0.9% NaCl, followed by i.p. injection with a saline solution of Rose Bengal (0.2 ml of 10 mg/ml in 0.9% NaCl). Illumination of the exposed skull with an optic fiber bundle connected to a cold light source (3200 Kelvin) led to a photochemical induction of Rose Bengal causing the formation of superoxide and singlet oxygen. This results in endothelial injury, platelet aggregation and activation, causing thrombosis of the cortical vessel in the region of the irradiated skull (Watson et al., 1985; Keiner et al., 2008, 2009).

### Infarct Volumetry Analysis

Using a charge-coupled device camera, Simple PCI software and Scion Image we measured the infarct area of the photothrombotic lesion (mm^2^) on every 8^Th^ cresyl violet stained section (containing approximately 6–7 slices). Further, the measured lesion areas of each animal were added and multiplied with the section interval and the section thickness (40 μm) to determine the lesion volume per animal and group.

### BrdU Administration

For proliferation studies, animals were i.p. injected with a single daily dose of Bromodeoxyuridine (BrdU, 50 mg/kg body weight, Sigma-Aldrich) on days 2–6 post-PIT. The number of BrdU-incorporating astrocytes was counted 14 days post-PIT. BrdU was dissolved in 0.9% NaCl and sterile filtered.

### Statistical Analysis

For statistical analysis, we first tested for normal distribution using the Shapiro–Wilk test. If normally distributed, significance levels were assessed using unpaired Student’s *t*-test with unequal variances, for non-normal distribution, the Mann–Whitney test was used. Differences were considered statistically significant at ^∗^*p* < 0.05, ^∗∗^*p* < 0.01, and ^∗∗∗^*p* < 0.001. All data are presented as mean ± SEM (standard error of the mean). The number of samples analyzed for each experiment is indicated in the figure legends.

## Results

### Astrocytes Expressed ETC and oxPhos Components *in vivo*

**FIGURE 1:**
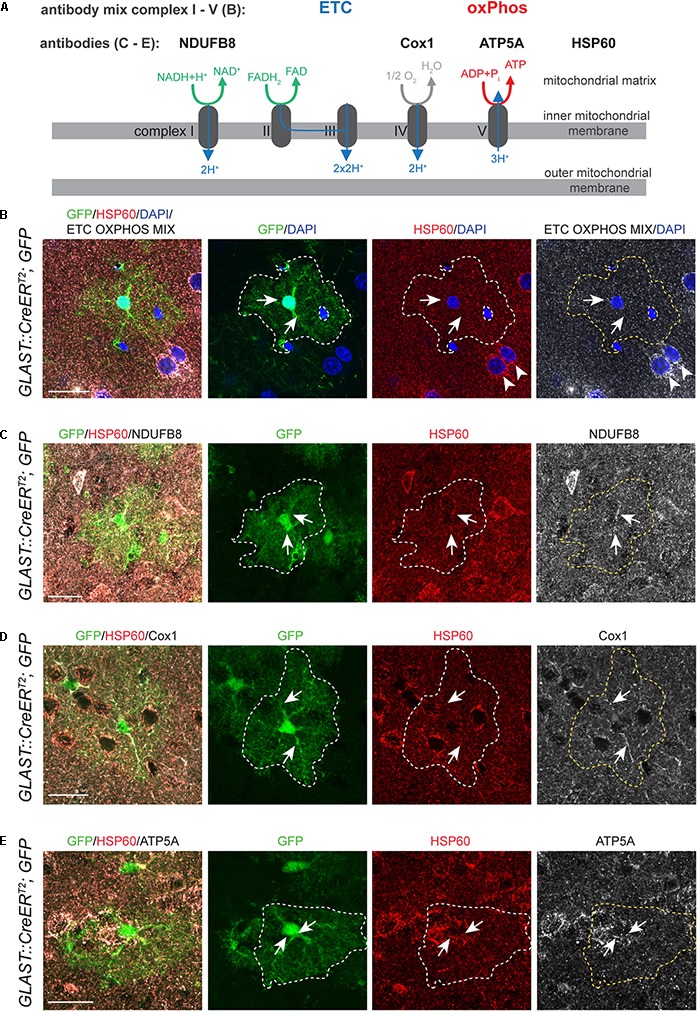
Astrocytes express ETC and oxPhos components *in vivo.* **(A)** Schematic drawing illustrating localization and function of mitochondrial complexes of the electron transport chain (ETC) and oxidative phosphorylation (oxPhos); antibodies used in **(B–E)** to visualize expression of mitochondrial complex components are indicated. **(B)** DAPI was used to counterstain nuclei of glial and neuronal cells (arrowheads). **(B–E)** *Glast::Cre^ERT2^;GFP* mice labeled single astrocytes (green; outlined by white or yellow dotted line in all pictures except merge on the left); HSP60 labeled mitochondria (red); immunoreactivity for ETC OXPHOS MIX **(B)** and specific mitochondrial complex components NDUFB8 **(C)**, Cox1 **(D)**, ATP5A **(E)** shown in white. Immunostainings revealed that astrocytes in the cortex express all mitochondrial complex components tested (arrows in **B–E**). Pictures represent collapses of 2–3 confocal stacks. All scale bars = 20 μm.

Data from mRNA expression analyses suggest that astrocytes express the necessary molecular machinery to perform oxidative metabolism (Lovatt et al., 2007; Cahoy et al., 2008). To further support this notion and to validate the expression of ETC and oxPhos components on protein level, we made use of a recently established fluorescent immunohistochemistry protocol for the labeling of mitochondrial matrix protein and components of the mitochondrial ETC and oxPhos complexes *in vivo* in brain sections (Beckervordersandforth et al., 2017). To visualize the astrocytic cell bodies, we crossed *GLAST::CreER^T2^* mice (Mori et al., 2006) to cytoplasmic GFP reporter mice [CAG CAT GFP mice; (Nakamura et al., 2006)]. Recombination was induced by intraperitonial administration of Tamoxifen at postnatal days 14, 16, and 18, and resulted in mosaic labeling of cortical astrocytes. Mitochondria were identified using an antibody against mitochondrial chaperone HSP60 (Figure 1A–E), an intramitochondrially localized molecule (Bukau and Horwich, 1998). First evidence for the expression of mitochondrial ETC and oxPhos components in cortical astrocytes came from the analysis of ETC/OXPHOS antibody-mix (Figure 1B, arrows), containing five different antibodies against components of mitochondrial complexes I–V. GFP-labeled cortical astrocytes revealed expression of ETC/OXPHOS components in HSP60^+^ mitochondria (Figure 1B, arrows). Surrounding cortical neurons contained a large number of HSP60^+^ mitochondria expressing the ETC/OXPHOS mix (arrowheads; Figure 1B). To delineate individual mitochondrial complexes we next used antibodies against single components of complex I (NADH: ubiquinoneoxidoreductase subunit B8, NDUFB8; Figure 1C), complex IV (Cytochrome c oxidase subunit 1, Cox1; Figure 1D) and complex V (ATP synthase subunit 5A, ATP5A; Figure 1E). All tested components were present in astrocytic mitochondria, indicating that cortical astrocytes express ETC and oxPhos proteins *in vivo*.

### Conditional Deletion of *Tfam* in Cortical Astrocytes Induced Reactive Gliosis and Mitochondrial Swelling

Next, we aimed to assess if cortical astrocytes require a functional ETC and oxPhos machinery. For that reason, ETC and oxPhos activity was genetically disrupted by conditional deletion of *Tfam*, a transcription factor required for the transcription of mitochondrial DNA, which mostly encodes for components of ETC and oxPhos complexes. By crossing *Tfam^*fl/fl*^* mice (Larsson et al., 1998) with *GLAST::CreER^T2^* and *CAG CAT GFP*, we generated conditional knockout mice that upon Tamoxifen-induced recombination lacked Tfam (*Tfam^cko^*), and as a consequence transcription of mtDNA encoded ETC and oxPhos components in astrocytes. GFP-reporter expression allowed for the detection of recombined astrocytes. *GLAST::CreER^T2^; CAG CAT GFP* mice harboring WT alleles for *Tfam* served as controls (*Tfam^ctrl^*). Recombination was induced postnatally as described above. Because of the long half-life of *Tfam* transcripts conditional *Tfam* mutants consistently show protracted development of a phenotype after disruption of the *Tfam* locus (Wang et al., 1999; Sorensen et al., 2001; Ekstrand et al., 2007; Beckervordersandforth et al., 2017). Analysis was therefore carried out starting 3 months post-induction. First, we validated if Cre-mediated recombination led to a loss of Tfam. To assess recombination of the *Tfam* locus *in vivo*, we isolated GFP-expressing astrocytes from different brain areas of postnatally recombined animals by fluorescent activated cell sorting (FACS), and performed PCR (Supplementary Figure 1A). Astrocytes isolated from *Tfam^ctrl^* mice revealed a single band of 404 bp representing the wildtype (Supplementary Figure 1B, left). In astrocytes isolated from the cortex of *Tfam^cko^* animals we detected a strong *Tfam^cko^* band (330 bp) and a weak *Tfam^*floxed*^* band (437 bp; Supplementary Figure 1B, right), indicating that the *Tfam* locus was successfully recombined *in vivo*. Due to an unreliable immunofluorescent signal of the available TFAM antibodies *in vivo*, we were not able to assess if recombination led to loss of Tfam protein. We therefore switched to an *in vitro* system, and isolated cortical astrocytes from postnatal day 5 mice harboring the GFP-reporter as well as conditional *Tfam^*floxed*^* alleles (*CAG CAT GFP; Tfam^*fl/fl*^*). Recombination was induced by transduction with the His-TAT-NLS-Cre (HTNCre) protein (Supplementary Figure 1C; Peitz et al., 2002). Six days post-transduction, the astrocytes were either fixed for immunohistochemistry (Supplementary Figure 1D), or used for PCR to validate recombination of the conditional *Tfam* locus (Supplementary Figure 1E). Immunohistochemistry against the Tfam protein revealed that Tfam was almost completely absent in GFP^+^ astrocytes, but present in non-recombined GFP^-^ astrocytes (Supplementary Figure 1D). Importantly, the mitochondrial marker Cytochrome C (CytC) confirmed that mitochondria were present in both non-recombined GFP^-^ and recombined GFP^+^ astrocytes (Supplementary Figure 1D). PCR of cortical astrocyte cultures transduced with increasing amount of HTNCre (1, 2, and 4 μl) revealed an increase of the *Tfam^cko^* band (330 bp), and a reduction of the *Tfam^*floxed*^* band (437 bp; Supplementary Figure 1E). These results indicated that the conditional *Tfam* locus could be efficiently recombined in cortical astrocytes by Cre recombinase. The results also confirmed that Tfam protein is lost upon Cre-mediated recombination, and further validated that recombination events correlated with the expression of the GFP reporter.

**FIGURE 2:**
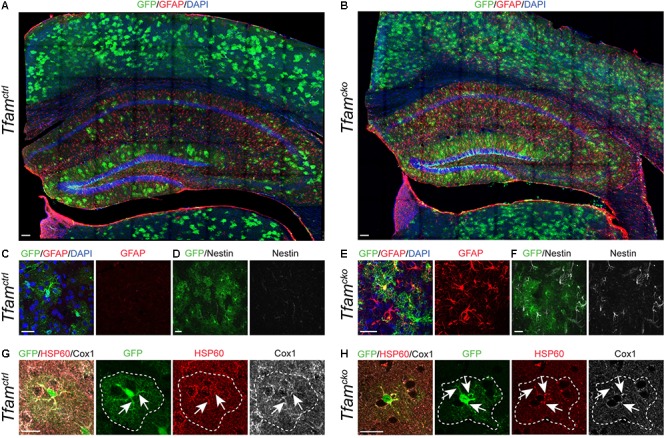
*Tfam* deletion in astrocytes induces reactive gliosis and aberrant mitochondrial morphology in the cortex. Phenotypic comparison between *Tfam^ctrl^* (left) and *Tfam^cko^* mice (right). **(A–F)** *Tfam* deletion in astrocytes induced reactive gliosis in the cortex as shown by intermediate filament markers GFAP **(A–C,E)** and Nestin **(D,F)**. **(G,H)** Immunostaining for *Tfam*-independent mitochondrial marker HSP60 (red) and *Tfam*-dependent mitochondrial protein Cox1 (white); GFP (green) served to identify recombined cells (outlined by white dotted lines). *Tfam* depletion led to mitochondrial clumping in astrocytes, but did not result in loss of Cox1 expression (arrows). All scale bars = 20 μm, except **(A,B)** scale bars = 100 μm.

We first investigated the effects of *Tfam* deletion in cortical astrocytes *in vivo*. In the cortex, *GLAST::CreER^T2^* driven recombination efficiency was more than 50% as reported before (Supplie et al., 2017), and was comparable between genotypes 4 months and 1 year post-recombination (Figure 2A,B and Supplementary Figures 2A,B), indicating that deletion of *Tfam* in astrocytes did not affect their survival. However, *Tfam* dysfunction induced upregulation of intermediate filament components glial acidic fibrillary protein (GFAP, Figure 2A–C,E) and Nestin (Figure 2D,F). Neither GFAP nor Nestin are expressed in astrocytes of the adult cortex under physiological conditions. Only upon challenge, such as injury or disease astrocytes become reactive, and upregulate GFAP and Nestin as an important hallmark of reactive gliosis (Robel et al., 2011). Thus, *Tfam* deletion induced a reactive gliosis in cortical astrocytes (Figure 2B,E,F) in comparison to *Tfam^ctrl^* mice (Figure 2A,C,D).

Next, we wanted to find out how *Tfam* deletion affected mitochondria of astrocytes. Therefore, analysis of mitochondrial morphology and expression of HSP60 together with ETC and oxPhos components in recombined cells in *Tfam^ctrl^* and *Tfam^cko^* animals was carried out (Figure 2G,H). While mitochondrial shape was undistinguishable between recombined and non-recombined cells in *Tfam^ctrl^* mice (Figure 2G), 55% of recombined cells in *Tfam^cko^* mice contained mitochondria with an aberrant clumpy morphology and increased HSP60 expression levels (Figure 2H). These morphological alterations (often referred to as mitochondrial “swellings”) have been reported across different cell types following *Tfam*-depletion, including neurons (Beckervordersandforth et al., 2017), epidermal stem cells (Baris et al., 2011), cardiac stem cells (Chung et al., 2007), and brown adipose tissue (Vernochet et al., 2012), and are indicative of mitochondrial dysfunction. Surprisingly, and in contrast to *Tfam* deletion in newborn neurons (Beckervordersandforth et al., 2017), mitochondria of recombined astrocytes still contained mtDNA-encoded Cox1 protein (Figure 2H, arrows), whose expression requires *Tfam*.

### Photochemically Initiated Thrombosis Worsened Mitochondrial Morphology in *Tfam*-Depleted Cortical Astrocytes of the Perilesional Area

**FIGURE 3:**
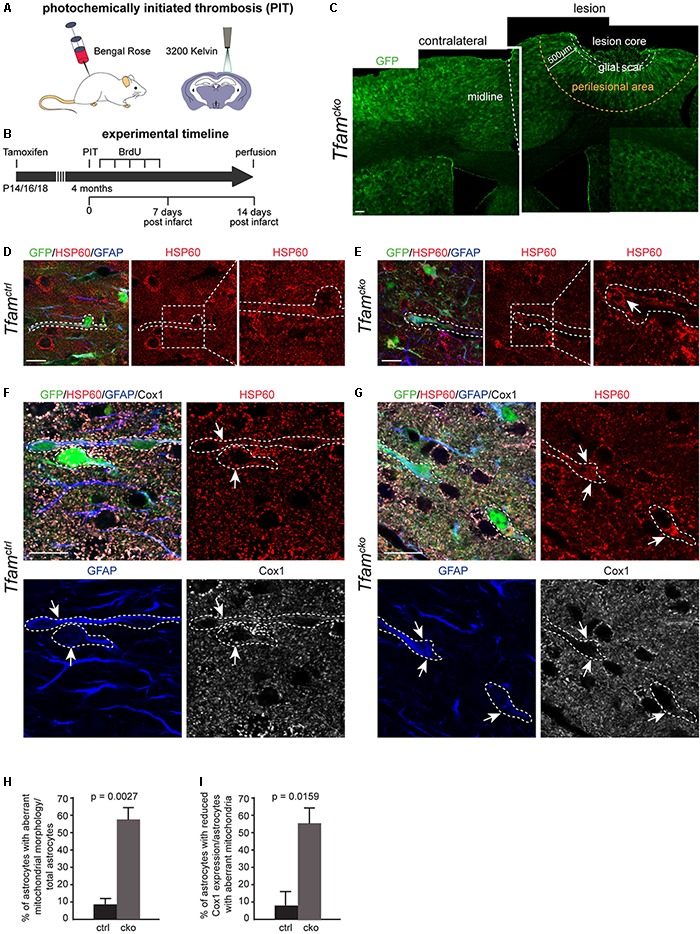
Photochemically initiated thrombosis (PIT) worsened mitochondrial phenotypes of *Tfam*-depleted reactive astrocytes and reduced *Cox1* expression. **(A,B)** Experimental scheme used in **(C–I)**. **(C)** Confocal image of a coronal cortical section of *Tfam^cko^* mice upon PIT; midline indicated by a white dotted line; GFP indicates recombined cells. Contralateral hemisphere shown on the left; PIT lesioned hemisphere (right) with glial scar (white dotted area) and perilesional area (yellow dotted area), which was defined as the area 500 μm away from the lesion. **(D–G)** Comparison of mitochondrial phenotype between *Tfam^ctrl^* and *Tfam^cko^* mice upon PIT (dotted lines represent process of recombined astrocyte); **(D,E)** dotted boxes indicate area of higher magnification on the right. **(D,F,H,I)** Few mitochondrial aberrations were detected in *Tfam^ctrl^* mice (**D**, arrows in **F**), while *Tfam* depletion led to mitochondrial elongation and hyperfusion, and diminished *Cox1* expression in reactive astrocytes (arrows; **E,G**). **(H)** Quantification of total astrocytes harboring aberrant mitochondria; **(I)** reduced Cox1 expression in perilesional astrocytes with aberrant mitochondria. **(D–I)** n_ctrl_ = 5 animals, n_cko_ = 5 animals. Data represent as mean ± SEM; *t*-test **(H)** and Mann-Whitney test **(I)** was performed to determine significance; scale bars = 100 μm **(C)** and = 10 μm **(D–G)**.

Changes in astrocyte function during injury response can markedly impair healing and regeneration of insulted CNS areas (Burda and Sofroniew, 2014). Next, we investigated if mitochondrial dysfunction impaired astrocytes function in the context of CNS injury. Ischemic stroke was induced in the cortex of 4 months old *Tfam^ctrl^* and *Tfam^cko^* mice by PIT (Figure 3A). The effects on reactive astrocytes and surrounding tissue were investigated 2 weeks later (Figure 3B). PIT created a lesion that affected all cortical layers but left the subcortical white matter intact (Figure 3C). The lesion consisted of three distinct regions as described before: core, glial scar, and perilesional area (Wanner et al., 2013; Burda and Sofroniew, 2014). The lesion core mainly contains dead cells and tissue that is irretrievably damaged. The glial scar consists of astrocytes and surrounds the core tissue as a structural border between dead non-neuronal tissue and viable cells. The perilesional area is adjacent to the scar and is populated by astrocytes, neurons, oligodendrocytes, and microglia (Wanner et al., 2013). It is the only part of the lesion responsive to interventions and rehabilitation therapies. Furthermore, astrocytes have been shown to play a role in plasticity of the perilesional area in response to PIT (Keiner et al., 2008). For these reasons, our analysis was focused on the perilesional area, which we defined as the area within 500 μm from the boarders of the lesion core (Figure 3C).

Traumatic brain injuries have been shown to induce changes in mitochondrial marker expression and ultrastructure in mice as well as in humans (Balan et al., 2013; Motori et al., 2013). Therefore, we first investigated if PIT-induced injury affected mitochondria of astrocytes by assessing their morphology (Figure 3D–H). In *Tfam^ctrl^* mice, a minor fraction of astrocytes in the perilesional area showed aberrations in mitochondrial morphology (8%; Figure 3H). The percentage of astrocytes harboring morphologically aberrant mitochondria did not change between *Tfam^cko^* animals in physiological and PIT-induced injury conditions (Figure 3H). While we mostly observed mitochondrial clumping in *Tfam^cko^* mice under non-injury conditions, in the perilesional area, *Tfam*-depleted reactive astrocytes came mainly in two flavors: a mitochondrial clumping phenotype as well as elongated mitochondria, which in some cells appeared as an interconnected meshwork of hyperfused organelles (Figure 3E,G). Interestingly, 56% of perilesional *Tfam* depleted astrocytes with aberrant mitochondria showed a reduction in Cox1-protein expression (Figure 3G,I). These results suggest that focal ischemia significantly worsened the mitochondrial phenotype of *Tfam*-depleted astrocytes as indicated by mitochondrial hyperfusion and a reduction in Cox1 expression.

### *Tfam* Dysfunction Impaired the Generation of New Reactive Astrocytes and Increased Death of Neurons in the Perilesional Area of PIT

**FIGURE 4:**
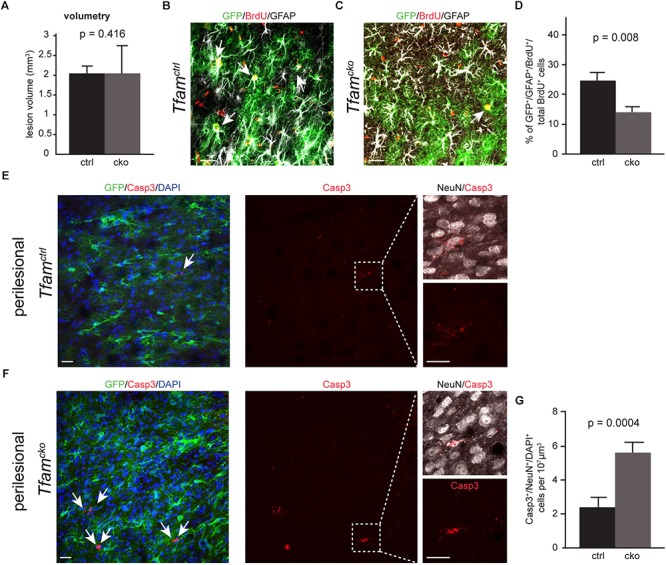
Mitochondrial dysfunction led to reduced generation of reactive astrocytes and increased neuronal death in the perilesional area. **(A)** Lesion volumetry revealed no difference between *Tfam^ctrl^* and *Tfam^cko^* mice. **(B–D)** Confocal picture of *Tfam^ctrl^* **(B)** and *Tfam^cko^* **(C)** animal stained against BrdU (red), GFP (green), and GFAP (white). **(D)** Percentage of GFAP^+^/GFP^+^ cells amongst all BrdU^+^ cells was sigificantly reduced in *Tfam^cko^* mice in comparison to *Tfam^ctrl^*. **(E–G)** Confocal images and quantification of Casp3 immunostaining (red) in *Tfam^ctrl^* and *Tfam^cko^* mice; GFP^+^ shows recombined cells (green); NeuN labels neurons (white); DAPI indicates cell nuclei. *Tfam* deletion in astrocytes significant increased neuronal cell death in the perilesional area **(G)**. **(A–D)** n_ctrl_ = 6 animals, n_cko_ = 8 animals; **(E–G)** n_ctrl_ = 4 animals, n_cko_ = 4 animals. Data represented as mean ± SEM; *t*-test was performed to determine significance; all scale bars = 20 μm.

Previous work has shown that the infarct size upon PIT is highly reproducible, which makes this model very suitable to investigate clinical outcome and repair mechanisms (Diederich et al., 2014; Frauenknecht et al., 2016). We therefore determined if *Tfam* deletion in reactive astrocytes changed lesion volumetry. Fourteen days after PIT, the infarct volume was comparable between *Tfam^ctrl^* and *Tfam^cko^* mice (Figure 4A), indicating that deletion of *Tfam* did not alter infarct size. Next, we wanted to know if *Tfam*-depletion affected survival of astrocytes upon PIT. Counting the number of recombined astrocytes in the perilesional area 2 weeks post-infarct revealed no changes between *Tfam^ctrl^* and *Tfam^cko^* mice (Supplementary Figure 2C), suggesting that also under severe injury conditions astrocytes are not dependent on mitochondrial respiration for survival.

A key parameter in injury-induced reactive gliosis is proliferation of reactive astrocytes. To investigate the proliferative response of astrocytes in *Tfam^*ctrl*^* and *Tfam^cko^* animals, we determined the number of newly generated astrocytes in the perilesional area. Animals received five consecutive injections of Bromodeoxyuridine (BrdU) on days 2–6 post-PIT, and the number of BrdU-incorporating astrocytes was counted 14 days post-PIT (Figure 3B). Here, we observed a significant decrease in BrdU incorporation of recombined reactive astrocytes in the *Tfam^cko^* mice compared to *Tfam^ctrl^* mice (BrdU^+^/GFAP^+^/GFP^+^/total BrdU^+^ cells; Figure 4B–D).

These data suggest that the generation of new astrocytes in the perilesional area is hampered in astrocytes with mitochondrial dysfunction.

Astrocytes have neuroprotective functions potentially through secretion of neurotrophic factors, the supply of metabolites and transfer of antioxidant molecules (Belanger and Magistretti, 2009). We next examined whether *Tfam*-deletion induced mitochondrial dysfunction compromised the neuroprotective role of astrocytes. To this end, we combined immunostaining against the apoptosis marker activated Caspase 3 (Casp3) with the neuronal marker NeuN, and quantified the number of Casp3^+^/NeuN^+^ neurons in the perilesional area (Figure 4E–G). In the contralateral hemisphere, we did not detect differences in the number of Casp3^+^ cells between *Tfam^ctrl^* and *Tfam^cko^* animals (Supplementary Figures 3A–F). Intriguingly, the number of Casp3^+^/NeuN^+^ neurons was more than doubled in *Tfam^cko^* compared to *Tfam^ctrl^* animals (Figure 4D–F). These results indicate that mitochondrial dysfunction in astrocytes led to increased neuronal cell death upon PIT, suggesting that mitochondrial dysfunction compromises the neuroprotective function of astrocytes.

## Discussion

The requirement and function of mitochondrial oxidative metabolism in astrocyte physiology are a matter of ongoing debate. Only recently, convincing *in vivo* evidence has been provided that a particular astrocyte subtype, i.e., Bergmann Glia of the cerebellum does not require ETC/oxPhos for long-term-survival (Supplie et al., 2017). Here, we investigated the *in vivo* requirement of ETC/oxPhos in cortical astrocytes in physiological and injury context. We demonstrate that cortical astrocytes express components of the ETC and oxPhos complexes. Genetically induced dysfunction of mitochondrial oxidative metabolism did not affect astrocyte long-term survival, but caused reactive gliosis in the forebrain. Upon challenge by PIT induced ischemic stroke, mitochondrial dysfunction compromised the response of reactive astrocytes as it was associated with a decrease in the generation of new astrocytes and increased neuronal cell death in the perilesional area. These results indicate that mitochondrial respiration is not essential for astrocyte survival but is required for reactive astrocyte function.

Our data extend the recent experimental evidence that ETC and oxPhos functions are not required for long-term survival from Bergmann glia to forebrain astrocytes. In the previous study, ETC/OxPhos deficiency caused by Cox10 ablation neither induced alterations in cerebellar cytoarchitecture nor caused reactive gliosis. In contrast, deletion of *Tfam* resulted in reactive gliosis in the cortex, as indicated by strongly upregulated intermediate filaments GFAP and Nestin. Why does deletion of *Tfam* induce such a strong glial phenotype, while no signs of glial pathology and neurodegeneration could be observed upon *Cox10* deficiency? The discrepancy between the two studies might be explained by the fact that *Cox10* dysfunction affects exclusively complex IV. *Tfam* deletion efficiently reduces the activity of complexes I, III, IV and V (Larsson et al., 1998; Baris et al., 2011; Vernochet et al., 2012), resulting in a more severe dysfunction of ETC and oxPhos as suggested by the aberrant mitochondrial morphology. The observed mitochondrial “swellings” in astrocytes are indicative of mitochondrial dysfunction and have been observed in various cell types upon *Tfam* deletion, including skeletal muscles, epidermal and cardial stem cells, adipose tissue, hepatocytes, Schwann cells, and neurons (Sorensen et al., 2001; Diaz et al., 2005; Baris et al., 2011; Funfschilling et al., 2012; Vernochet et al., 2012; Beckervordersandforth et al., 2017). Furthermore, in the context of neurodegenerative diseases, especially ETC complex I appears to play a crucial role in development of pathological symptoms (Mizuno et al., 1989; Schapira et al., 1989; Papa et al., 2002; Keeney et al., 2006; Cassina et al., 2008), supporting the idea that impairment of multiple complexes, including complex I, may have more severe consequences on astrocyte function than the deletion of complex IV alone.

Reactive gliosis is a finely graded process ranging from mild astrogliosis with potential for resolution to an extreme form involving scar formation. Severe tissue damage, including stroke, lead to proliferation of reactive astrocytes and to formation of a compact glial scar surrounding the lesion and separating the damaged non-functional lesion core from adjacent and potentially functional neural tissue (Faulkner et al., 2004; Gadea et al., 2008; Sirko et al., 2013; Wanner et al., 2013). Transgenic disruption of the astrocyte scar by ablation of proliferating astrocytes lead to increased death of local neurons, and impaired recovery of function after focal insult (Faulkner et al., 2004; Herrmann et al., 2008; Li et al., 2008; Wanner et al., 2013). Our work uncovered a potential link between mitochondrial function and the generation of new astrocytes by showing that ETC and oxPhos dysfunction significantly reduced BrdU incorporation of astrocytes in the perilesional area in a PIT-induced stroke model suggesting a proliferation defect of *Tfam*-deficient reactive astrocytes. *Tfam*-deficiency may have also lead to impaired survival of astrocytes generated in response to injury. Immunohistochemistry against Casp3 showed that at 14 days post PIT-induced stroke astrocytic cell death is a very rare event in both *Tfam^ctrl^* and *Tfam^cko^*. Further experiments are required to establish whether *Tfam*-deficiency may affect astrocytes survival in the PIT-induced stroke context at other time points.

How can mitochondrial respiration influence proliferation? One possible explanation may be that ETC is especially important in dividing cells because it accepts reducing equivalents generated during pyrimidine nucleotide synthesis and is therefore necessary for RNA- and DNA- production. Astrocytes in the cortex are post-mitotic under healthy conditions and not affected by the lack of Tfam. Upon injury, they are forced to divide and to generate new DNA building blocks. This process may be hampered by the accumulation of reducing equivalents, which are normally recycled by functional ETC complexes.

Under injury conditions, the mitochondrial phenotype of perilesional astrocytes deteriorated from swelled/clumped mitochondria to a hyperfused mitochondrial network. Mitochondrial fusion is essential for preserving quality control and intermixing of mitochondrial contents to facilitate changes in respiratory capacity, and adapt to cellular metabolic demands. Interestingly, it has been shown that mitofusin 2 (Mfn2), the key protein in mitochondrial fusion (Ehses et al., 2009; Belenguer and Pellegrini, 2013), negatively regulates cell proliferation. In an *in vitro* model of reactive gliosis, Mfn2 overexpression inhibits proliferation of reactive astrocytes by arresting the transition of cell cycle from G1 to S phase (Liu et al., 2014). Based on these results, it is tempting to speculate that proliferation may be blocked by cell cycle arrest in *Tfam*-depleted reactive astrocytes. It will be very interesting in the future to investigate the link between proliferation capacity and mitochondria dynamics.

*Tfam*-deletion in astrocytes of the perilesional area led to increased cell death of surrounding neurons. Astrocytes have the potential to enhance neuronal survival and regeneration through the release of, e.g., metabolic factors, detoxifying molecules, and neurotrophic factors (Sofroniew and Vinters, 2010; Allaman et al., 2011; Barreto et al., 2011; Cabezas et al., 2012). It is also becoming increasingly clear that protecting mitochondria in astrocytes is a promising strategy against loss of neurons in brain injury (Cabezas et al., 2012). An interesting candidate to link mitochondrial function in astrocytes to their role in neuroprotection is the translocator protein 18 kDA (TSPO). TSPO is located at the outer mitochondrial membrane and transports cholesterol into the inner compartments of mitochondria, which is the rate-limiting step in neurosteroid synthesis (reviewed in Milenkovic et al., 2015; Papadopoulos et al., 2018). Neurosteroids are important factors for protecting the brain from damage induced by, e.g., ischemic stroke or neurodegeneration (Tajalli-Nezhad et al., 2018). Interestingly, TSPO expression as well as binding of its ligands are upregulated in stroke and neurodegenerative disorders, implicating a regulatory role of mitochondrial TSPO in neurosteroid-mediated neuroprotection in pathological states. For future research, it will be very interesting to investigate if TSPO function and neurosteroid-dependent neuroprotection are affected by mitochondrial dysfunction. With the aim to identify new strategies to regenerate brain tissue after injury, a detailed understanding of the critical mitochondrial metabolic circuits in astrocytes and their function in neuroprotection is required.

## Data Availability Statement

All relevant data is contained within the manuscript.

## Author Contributions

CF, BE, RJ, DL, and RB: conceptualization. CF, SK, BE, IS, and RB: investigation. CF, SK, BE, and RB: formal analysis. RB: resources and funding acquisition and writing-original draft. All authors contributed to manuscript revision, read and approved the manuscript.

## Conflict of Interest Statement

The authors declare that the research was conducted in the absence of any commercial or financial relationships that could be construed as a potential conflict of interest. The reviewer FC and handling Editor declared their shared affiliation at the time of review.
