# Supplementary material for: Mitochondrial Dysfunction in Astrocytes Impairs the Generation of Reactive Astrocytes and Enhances Neuronal Cell Death in the Cortex Upon Photothrombotic Lesion
Source: Front Mol Neurosci. 2019 Feb 22;12:40. doi: 10.3389/fnmol.2019.00040 (PMC6395449; doi:10.3389/fnmol.2019.00040)
Supplement: Supplementary file 1 [file Data_Sheet_1.pdf]

Supplemental information

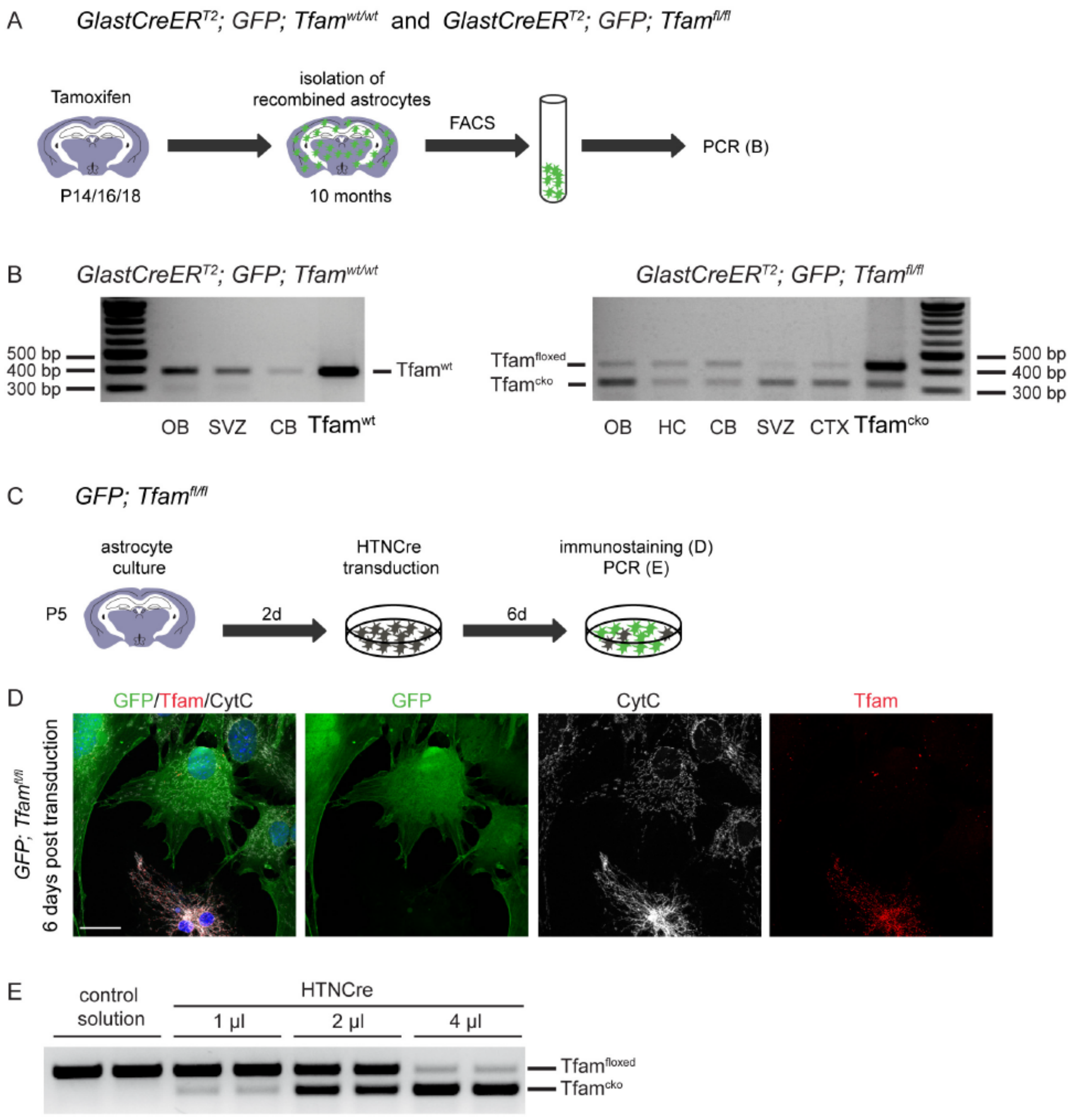

Supplemental Figure 1: Confirmation of *Tfam* deletion in astrocytes
(A) Experimental paradigm used for (B). (B) Genotyping PCR of GFP-expressing astrocytes isolated by FACS from distinct brain regions of 10 months old *Tfam*^{ctrl} and *Tfam*^{cko} mice (3 animals per genotype). *Tfam*^{ctrl} animals exclusively carried the *Tfam*^{wt} band (404 bp); positive control (Tfam^{wt} lane) was DNA isolated from tail clips of *Tfam*^{ctrl} mice. Recombination of the *Tfam* locus in astrocytes isolated from diverse brain regions of *Tfam*^{cko} mice was indicated by *Tfam*^{cko} band (330bp); *Tfam*^{floxed} band (437bp); DNA of *Tfam*^{cko} lane derived from tail clips of *Tfam*^{cko} mice. OB = olfactory bulb, SVZ = subventricular zone, CB = cerebellum, HC = hippocampus, CTX = cortex. (C) Experimental scheme used in (D-E). (D) Confocal image of HTNCre transduced astrocytes derived from *GFP; Tfam*$^{fl/fl}$ animals; Immunohistochemistry against the GFP reporter (green) indicates recombined cells; Cytochrome C (CytC, white) labels mitochondria; recombined astrocytes (GFP^{+}) lost expression of Tfam (red); non-recombined astrocytes (GFP^{-}) expressed Tfam. (E) Genotyping PCR of astrocytes transduced

with different amounts of HTNCre or control solution. Increasing recombination efficiency of the *Tfam* locus occurred with increasing concentration of Cre protein. Band size: $Tfam^{floxed}$ band = 437bp, $Tfam^{cko}$ band = 330bp. Scale bar = 10µm.

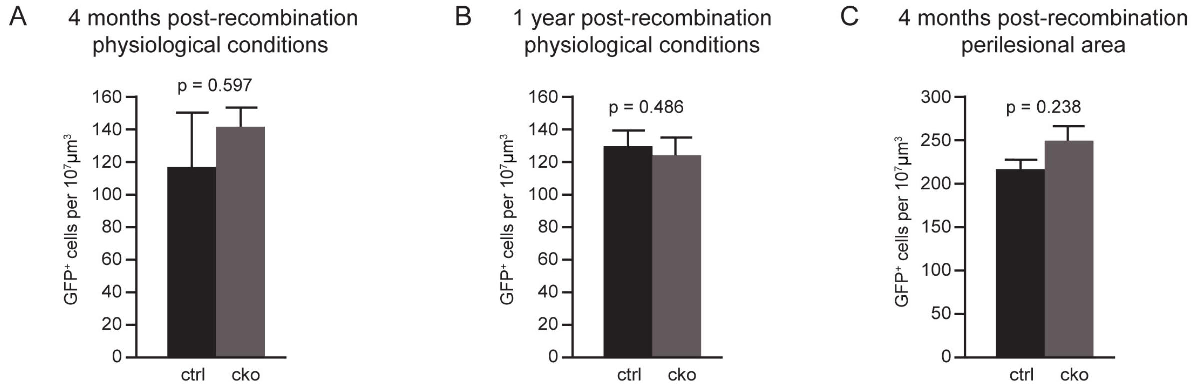

Supplemental Figure 2: *Tfam*-deficient astrocytes survive under physiological and injury conditions

(A-C) Survival of astrocytes in $Tfam^{ctrl}$ and $Tfam^{cko}$ mice was measured by counting recombined astrocytes (A) under physiological conditions 4 months post-recombination, (B) one year post-recombination; (C) upon PIT-induced injury 4 months post-recombination. (A) n_{ctrl} = 3 animals, n_{cko} = 4 animals; (B) n_{ctrl} = 4 animals, n_{cko} = 4 animals; (C) n_{ctrl} = 5 animals, n_{cko} = 5 animals. Data represented as mean ± SEM; t-test (B, C) and Mann-Whitney test (A) were performed to determine significance.

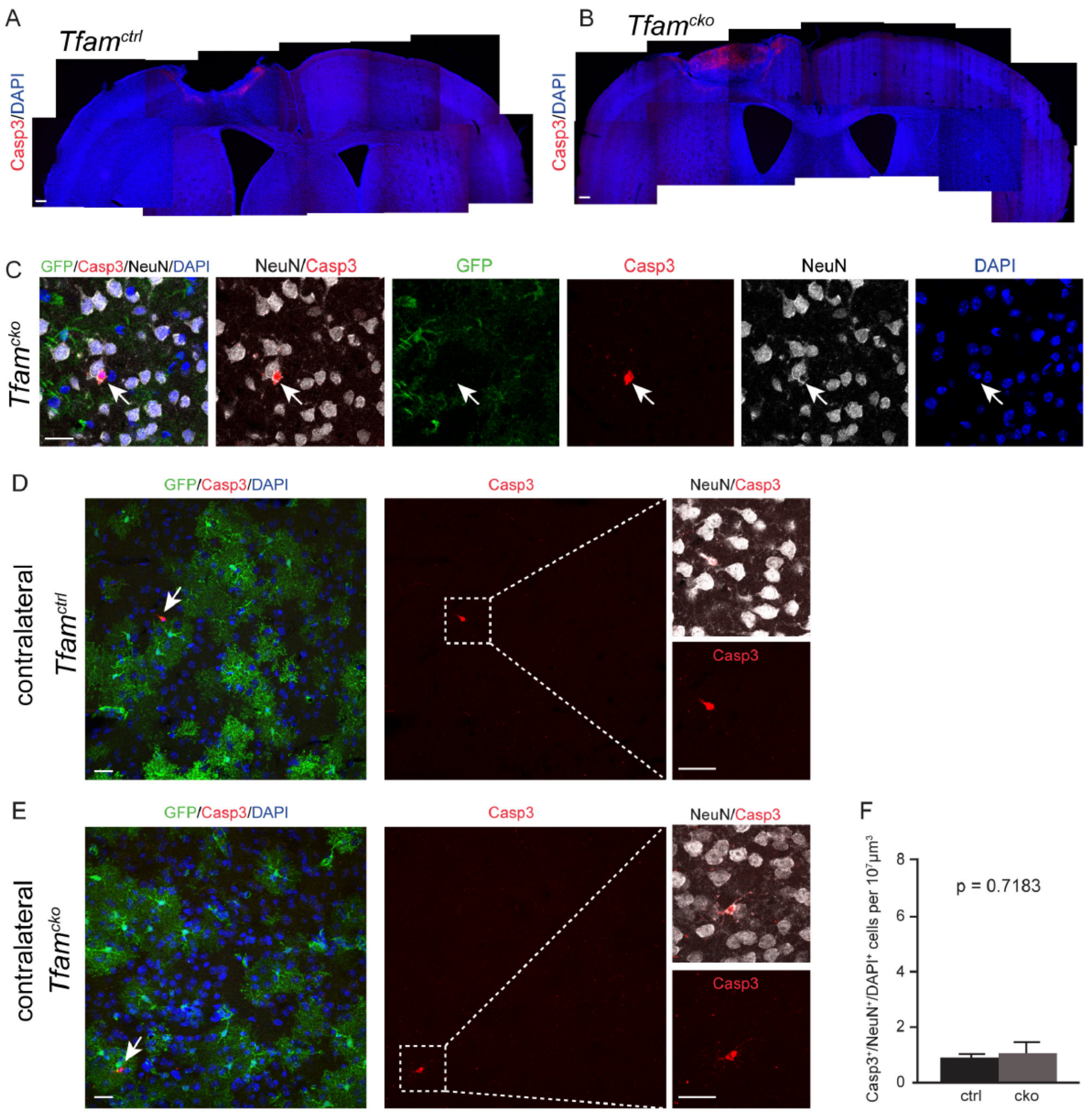

Supplemental Figure 3: Cell death in the contralateral and PIT-lesioned cortex

(A-B) Confocal images of a coronal cortical section from $Tfam^{ctrl}$ and $Tfam^{cko}$ mice upon PIT; immunohistochemistry against Casp3 (red) to identify dying cells; nuclei stained with DAPI (blue). Contralateral hemisphere shown on the right; PIT lesioned hemisphere (left) with lesion core containing $Casp3^{+}$ cells and damaged tissue. Very few cell undergo cell death in the contralateral hemisphere. (C) High magnification confocal image of perilesional area; immunostaining against $Casp3^{+}$ in red (cell death marker; arrows); GFP (labeling recombined cells, green); $NeuN^{+}$ (neuronal marker; white), and DAPI (nulcei; blue) showing a dying neuron ($Casp3^{+}$/GFP^{-}/$NeuN^{+}$). (D-F) Confocal images and quantification of Casp3 immunostaining (red) of the contralateral hemispheres; GFP^{+} shows recombined cells (green); NeuN labels neurons (white); DAPI indicates cell nuclei. No difference in neuronal cell death was detected contralaterally between $Tfam^{ctrl}$ and $Tfam^{cko}$ mice (F). (D-F) n_{ctrl} = 5 animals, n_{cko} = 5 animals. Scale bars = 100µm (A-B), and = 20µm (C-E). Data represented as mean ± SEM; t-test was performed to determine significance.
